# Regulatory Reform Outcomes and Accelerated Regulatory Pathways for New Prescription Medicines in Australia

**DOI:** 10.1007/s43441-022-00465-2

**Published:** 2022-10-21

**Authors:** Alina Yoffe, Johnson Liu, Greg Smith, Orin Chisholm

**Affiliations:** 1 Department of Pharmacology, School of Medical Sciences, Faculty of Medicine and Health, UNSW Sydney, Sydney, NSW Australia; 2 Sydney Pharmacy School, Faculty of Medicine and Health, The University of Sydney, Sydney, NSW Australia

**Keywords:** National Regulatory Authority, Regulatory affairs, Facilitated pathways, Early access, TGA, Recognition, Reliance

## Abstract

National Regulatory Authorities (NRAs) globally are facing the challenge of evaluating pharmaceutical products in a speedy manner, whilst simultaneously ensuring adequate efficacy, safety and quality of approved products. Additionally, common expectations include that the evaluation process is competent, flexible, commensurate with risk, efficient and rapid. In 2014, the Australian regulatory system was out of step with global regulatory developments which led to a comprehensive regulatory review and reform process. As part of the reforms, two Facilitated Regulatory Pathways (FRP) were developed for prescription medicines: Priority Review (PR) and Provisional Approval (PA). Furthermore, regulatory reliance and recognition arrangements have been expanded with the Therapeutic Goods Administration (TGA) making increased use of evaluation reports by trusted NRAs. The new pathways have been utilised by the pharmaceutical industry in Australia since 2017, with the number of medicines going through these pathways gradually increasing. Additional facilitated pathways have been developed following the review, providing alternatives to the standard pathway for registration of prescription medicines in Australia. The reform is timely, helping to position Australia well in the current global regulatory climate.

## Introduction

The Australian National Regulatory Authority’s (NRA), the Therapeutic Goods Administration (TGA), regulatory framework provides that medicines must meet safety and efficacy requirements to be authorised for marketing, thus protecting the public. This framework should also balance such considerations of safety, quality and efficacy with optimising market access to maximise the benefit of patients and industry, at the same time being in alignment with the government’s strategic goals and commitments, including productivity, innovation and competitiveness. Since October 2014, a significant regulatory reform has taken place in Australia, aiming to increase international regulatory alignment and collaboration, allow timely access to novel medicines and create a more flexible regulatory system capable of responding to rapid innovation in scientific and therapeutic product development [1].

In 2014 Australia didn’t have an accelerated review registration option, unlike the US or European Union, where such FRPs were available and utilised. As a direct outcome of the reform, two FRPs were introduced in Australia, which are the main focus of this review. As such, this paper examines the context and outcomes of the reform so far, with an emphasis on the regulation of prescription medicines (PM), in particular, New Chemical Entities (NCE) and new indications. These regulatory changes will make Australia a more desirable destination for regulatory submissions, have a positive influence on the international standing of the Australian Therapeutic Goods Administration (TGA), and ultimately result in improved medicine registration processes and public health benefits for Australians.

## Reform of Australian Regulatory System

### The Review Process

Australia’s regulatory frameworks for medicines had been in place for around 25 years by 2014, and the regulatory system was largely prescriptive rather than outcomes-based. In 2014, there was only one pathway utilised for the registration of new prescription medicines in Australia: the standard pathway. The legislated timeframe for evaluation of new prescription medicines via this pathway is 255 TGA working days and the pathway entails a full regulatory review of the dossier by the TGA. In 2014, TGA ranked fourth of the six major NRAs in terms of New Active Substances median time to approval, based on 2004–2013 data. The TGA median approval time was 391 days, compared with Swissmedic 511 days and FDA, the fastest NRA, with 304 days to approval [2]. In 2014, other jurisdictions had introduced facilitated regulatory pathways to ensure timely availability of new prescription medicines to their populations. Therefore, on 24 October 2014, the Australian Federal Government announced an independent review of Medicines and Medical Devices Regulation (MMDR). An expert panel was appointed consisting of Emeritus Professor Lloyd Sansom AO (Chair), Professor John Horvath AO and Mr Will Delaat AM [3]. Therapeutic Goods Act 1989 (Cth) and Therapeutic Goods Regulations 1990 are the regulatory legislative instruments, and some of the changes proposed by the review would affect this legislation.

**Figure 1:**
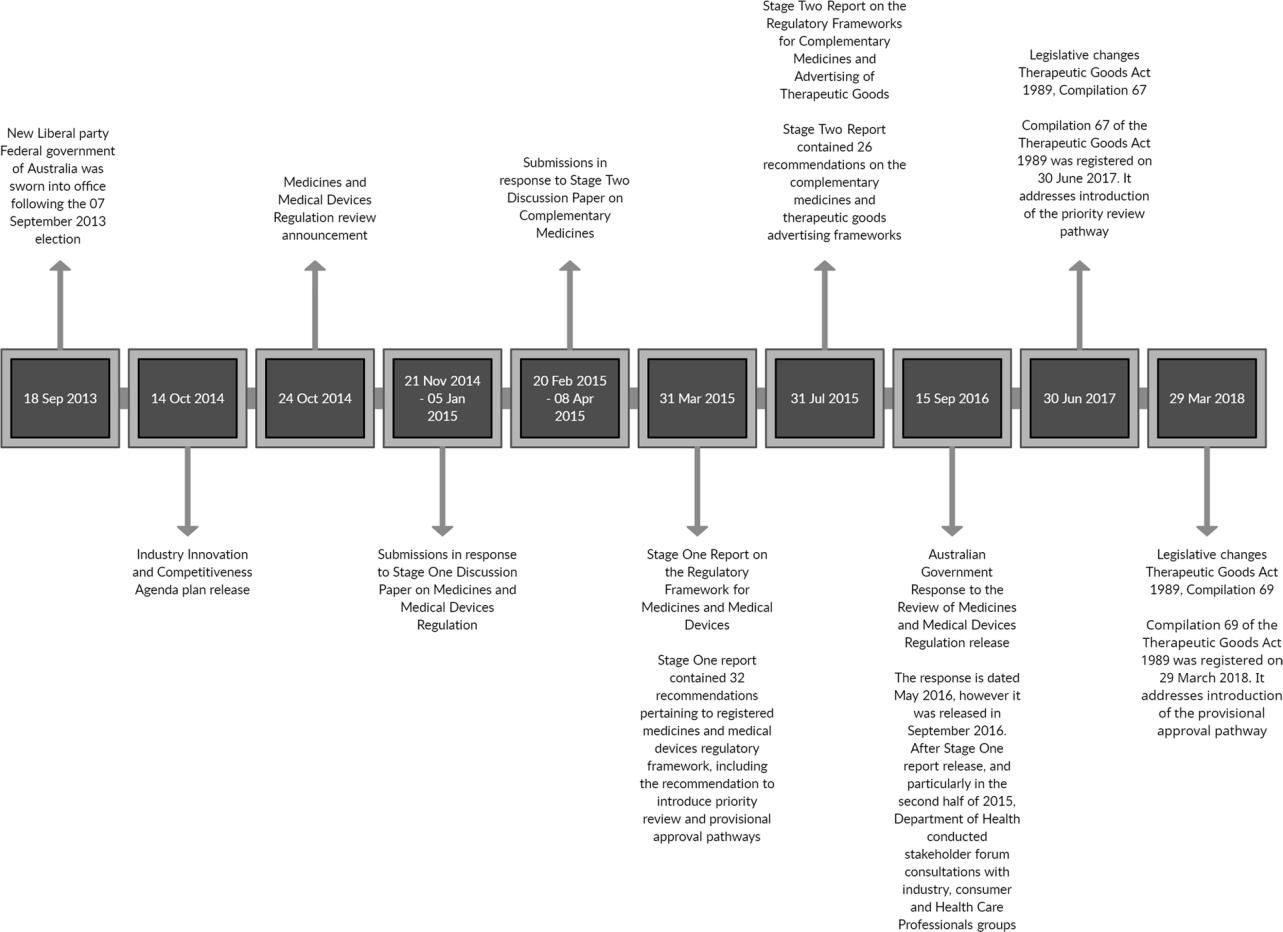
Medicines and Medical Devices Regulation review timeline [9, 21, 27, 37, 38]

The MMDR review process and methodology are described in detail on the Australian Government Department of Health (DoH) and TGA websites [4, 5]. The medicines regulation review was conducted in two stages: stage one included prescription and over the counter (OTC) medicines and medical devices; stage two included complementary medicines. This article will focus only on the review process and outcomes pertaining to prescription medicines. The review of PM regulation was completed in 5 months and the panel’s report was delivered to the Government on 31 March 2015 [4]. The review process outline and timeline are detailed in Fig. 1. The MMDR review aimed to remove inefficient and duplicative regulation, increase flexibility, simplify compliance, reduce costs to business, align with other NRAs and boost competitiveness of the Australian regulatory system, whilst preserving quality, safety and efficacy of medicines and medical devices [3]. The review covered different phases of a product lifecycle, including development, manufacturing, regulation and marketing [6]. It examined pharmaceuticals, medical devices, over the counter and complementary medicines and addressed patient access to unapproved therapeutic goods [2].

In Australia, the National Medicines Policy (NMP) was first published in the year 2000, providing a high-level policy framework for access to medicines, their use, quality, safety and efficacy standards [7]. Whilst NMP is not defined in the legislation, it guides the medication management activities, and enables appropriate structures and processes for equitable and timely access to safe and affordable medicines and medicines-related services. The key principles underpinning the NMP are timely access to medicines, at a price affordable to both the consumer and the community, Quality Use of Medicines, acceptable standards of safety, efficacy and quality, and pharmaceutical industry sustainability and responsible conduct [7]. The MMDR was conducted within the NMP framework, and its outcomes were bound to be consistent with it (Fig. 1).

### The Status Quo Prior to the Reform

The MMDR review stage one discussion paper evaluated the status quo of the regulatory system as of 2014, as well as stakeholders’ understanding of the issues and desired future developments. A short response submission period of 6.5 weeks was provided, from 21 November 2014 to 5 January 2015 [8]. During this time comprehensive input was collected via face to face and teleconference meetings, and 103 formal submissions received, representing a wide range of stakeholder views. Approximately half of the respondents were from industry and industry peak bodies, followed by just under 20% from health professionals/health professional bodies and consumers/consumer bodies. Academics, private health insurers and advisory groups also contributed submissions.

Stakeholders and the review panel highlighted several areas for improvement, including two important concerns: lack of an accelerated review option in contrast to other developed NRAs and lack of flexibility in making the assessment commensurate with risk, adequate for the type of product approved or the international approval status. As such, in 2014, the TGA undertook a complete de novo evaluation of NCEs, regardless of approval status by another NRA, resulting in duplication of regulatory efforts and processes. Extensions of indications were subject to the same timeframe as a de novo assessment. Furthermore, the TGA was the only NRA amongst comparable regulators with no accelerated or adaptive approval pathway options, therefore lacking the necessary flexibility to prioritise vital products for unmet needs [2]. The same legislated timeframe applied for medicines indicated for non-debilitating conditions, or not first in class, as applied to lifesaving and novel products.

By comparison, in the USA the availability of four FRPs enabled shortened Food and Drug Administration (FDA) evaluation times for certain products. The FDA has consistently made the widest use of the FRPs compared with other regulators and has achieved the shortest time to approval amongst leading NRAs. As such, between 2016 and 2020 the US FDA approved the highest proportion of drugs via FRPs (67%). In 2020, the FDA average time to approval of new active substances was 244 days (calculated for medicines in all pathways, from submission until approval, inclusive of NRA and company time) [9]. In Europe, accelerated review pathways were established in 2004 under Regulation (EC) No 726/2004 [10], whilst Priority Medicines (PRIME) has been available since 2016 [11] and conditional marketing authorisations since 2006. The EMA has consistently made low use of FRPs, with only 9% usage in 2020 and an average time to approval of 426 days [9]. Whilst the use of FRPs is not the only factor affecting review timelines, it has been the cornerstone of the NRA’s mechanisms to expedite approval.

### The Review Panel’s Recommendations

The stage one report on the regulatory framework for medicines and medical devices was released on 31 March 2015 and contained 32 recommendations [9]. Of these, two recommendations were related to the role of the regulator, 14 to the medicines’ regulatory framework, nine to the medical device framework, three to access to unapproved therapeutic goods and six to regulator functioning and good regulatory framework enablers. Fifteen of the thirty-two recommendations were pertinent to prescription medicines registration.

Whilst the panel viewed the TGA as a highly regarded and skilled NRA, it proposed several ways to improve the regulatory system and enhance TGA performance. As such, the panel referred to the Centre for Innovation in Regulatory Science (CIRS) data showing the TGA’s median approval time of 391 days in 2013—fourth out of the six regulators assessed: FDA (USA), Pharmaceuticals and Medical Devices Agency (PMDA, Japan), Health Canada, EMA (EU) and Swissmedic (Switzerland) (median approval time 304, 342, 350, 478 and 511 days, respectively) [12]. The TGA’s slower speed of review, combined with a lag in submission dates for registration between Australia and overseas regulators, resulted in delayed access to therapeutic goods for Australian citizens. Speeding up the TGA review could positively affect timing and/or readiness of companies to submit products for registration in Australia, particularly given the trend for simultaneous submissions to major NRAs.

The panel recommended that the TGA retain its capacity to undertake complete de novo evaluations and benefit-risk assessments across the entire review spectrum, particularly within the Australian context, including unique population groups, clinical and prescription practices and climatic conditions affecting the supply chain [2, 13]. They also proposed three registration pathways for pre-market assessment of prescription medicines.

The first pathway proposed was retaining the full de novo assessment pathway. However, the panel recommended work sharing for simultaneous submissions to optimise regulatory burden and further expedite review. Australia was well positioned, having existing involvement in international work sharing initiatives and pilot collaboration efforts, to pursue this option [2].

The second pathway proposed was for medicines which had been approved for marketing overseas. This pathway had been available under the previous TGA system but was not utilised by industry due to the requirement for multiple unredacted NRA reports, which were difficult to obtain. The proposed pathway would require a single un-redacted overseas approval report and broaden the list of trusted NRAs whose scientific rigour, track record, skills and quality of reports could be considered comparable to the TGA’s. The data package would need to be identical to the one approved overseas. Where a parameter was varied, the TGA would evaluate the different characteristics. The panel suggested that the reliance on overseas assessment should be counterbalanced by enhanced post-market monitoring [2].

The third option concerned expedited approval of a medicine under certain circumstances. The panel proposed the introduction of two FRPs, namely, priority review (PR) and provisional approval (PA), which were aligned with leading regulators’ practice yet tailored and optimised for Australia. In common with overseas FRPs, the Australian eligibility criteria would address lifesaving medicines and products which significantly improved quality of life (QoL), with consideration of seriousness of the disease, available alternatives and degree of innovation of the medicine [2]. A timeframe of 150 working days was proposed for PR, in line with the FDA’s 6 months (Priority Review) and the EMA’s 150 days (Accelerated Assessment). For PA the panel recommended a limited approval period, conditional on provision of comprehensive clinical data. The approval could be granted with limitations, such as a narrow patient population. Clear and comprehensive information for physicians and consumers as to the status of the medicine and its implications would be mandatory [2]. This recommendation was consistent with the Consumers Health Forum of Australia submission requesting better consumer education on regulatory schemes and their implications for medicine safety [14].

### Adoption of the Recommendations by the Government

Twenty three out of the 32 recommendations were accepted, including all recommendations pertaining to the role of the regulator and medicines regulatory framework. Of the recommendations related to regulator functioning and criteria for a good regulatory framework, namely recommendation 27 to 32, none were accepted in their entirety. Three were accepted partially, in principle, or the government stated it was supportive of intent. Two were rejected and one deferred [15]. The specific recommendations, and their adoption status are outlined in Table 1.

**Table 1:** MMDR Panel Recommendations and Government Adoption Status

Recommendation Number	Status	Summary
*Recommendations related to the role of the Regulator, TGA*		
1,2	Accepted	TGA will continue maintaining the capacity and necessary level of expertise to conduct full therapeutic goods assessments Australian government will retain the decision-making responsibility as to the inclusion of goods on ARTG
*Recommendations related to registration of NCEs*		
3,5,6,8,9,10,14	Accepted	A more useable pathway 2 is to be introduced for registration of an NCE previously approved overseas, including provision of a single non-redacted report and developing transparent and broader criteria for identification of “comparable” overseas regulatory agencies. Where the required materials used for overseas approval are provided to TGA, the regulator makes the decision within the Australian context, undertaking only the assessments, and only to a degree necessary to ensure safety, quality and efficacy within Australian population Introduction of PR and PA. The PA pathway has approval period limitation, data provision obligations, conditions imposed by TGA and mandatory advice to HCPs and consumers
*Recommendations related to regulator functioning and criteria for a good regulatory framework*		
27	Accepted except item 2	Post market monitoring is to be enhanced via analysis and integration of matched, de-identified datasets, improved Adverse Event awareness and reporting mechanisms. The government agreed in principle that organisational changes would be required to facilitate such improvements. Changes would also be required to improve integration of registration with HTA of a product Recommendation 28 to re-draft Therapeutic Goods Act 1989 was accepted in principle, however the implementation would be gradual with subsequent revision in the future as to the necessity of further changes to existing legislation The government rejected the idea of delegating registration decision making exclusively to the government’s Chief Medical Officer, with the support of a relevant advisory committee Designated government funding, in addition to the existing cost recovery model, has been deferred until future budget discussion and review
28	Accepted in principle	
29	Rejected	
30	Rejected	
31	Supportive of the intent	
32	Deferred	

Implementation of the reforms was staged over 3 years. Government adoption of the proposed pathways has resulted in the continuance of the de novo regulatory (standard) pathway, the introduction of a modified reliance pathway and the introduction of two FRPs. The Australian applicable legislation instruments, the Therapeutic Goods Act 1989 (Cth) and Therapeutic Goods Regulations 1990, have undergone necessary changes by adding compilations to facilitate the introduction of these new pathways.

Compilation No. 67 of the Act, registered on 30 June 2017, introduced a shorter review time for priority medicines, compared to the standard evaluation pathway [16]. The TGA target timeframe for approval of priority medicines is 150 working days, excluding the time required for the sponsor to answer TGA questions, although the legislative timeframe has not changed and is the same as the standard pathway, 255 working days. The Therapeutic Goods Regulations 1990, Compilation 77 Part 3C, registered on 11 July 2017, specified the requirements for PR applications and determination criteria. Priority determination may be granted to either a new medicine or a new indication, where there is a major therapeutic advance and where no alternative products are registered for the condition, or the priority product is a substantial improvement in safety or efficacy over existing approved products. PR determinations are valid for 6 months by which time the sponsor is expected to submit their full dossier for review. No specific recall conditions were introduced for medicines approved via PR pathway [16, 17].

For listing of provisionally approved goods a separate part was added to the Australian Register of Therapeutic Goods (ARTG), as specified in Compilation No. 69 of the Act, registered on 29 March 2018. The ARTG is a publicly accessible database of products which can be lawfully marketed in Australia. Given their inherent higher uncertainty, the Act specifies that the regulator may have a greater say in conditions of registration for provisionally approved products. As such, modifications may be made to the class of persons within an indication, instructions for use, warnings and precautions [16]. To facilitate the developing nature of the PA application, more flexible determination arrangements were allowed, with a six-month initial validity period, followed by a single extension for a further 6 months. Similar to priority products, the provisional determination may be granted to either a new medicine or a new indication where no alternative products are registered for the condition or when preliminary data shows that the provisional product constitutes a substantial improvement in safety or efficacy. Preliminary data must also indicate a major therapeutic advance. The sponsor must commit to submit comprehensive safety and efficacy data within a maximum period of 6 years after registration to convert the provisional registration to a full registration. At that time, the provisional registration lapses and the product will no longer be listed on the ARTG. In Australia, registration on ARTG is required to market a product. It is a pre-requisite for reimbursement and is separate to the reimbursement arrangements.

Compilation No. 80 of the Regulations, registered on 17 January 2018, introduced the timeframes for Comparable Overseas Regulator (COR)-A and COR-B review processes, i.e. 120 and 175 days, respectively, depending on an application meeting certain conditions for these pathways [17]. The COR pathways are discussed in further details below.

## Current Registration Pathways in Australia

Regulatory reform has continued to evolve since the adoption of the MMDR review recommendations in Australia. The TGA has worked with comparable NRAs to develop work-sharing schemes and other reliance mechanisms to facilitate earlier access to novel prescription medicines for the Australian population [18, 19].There are currently seven pathways available to sponsors of prescription medicines in Australia, outlined below: Standard pathway, Priority Review, Provisional Approval, Comparable Overseas Regulator (COR)-A, COR-B and two worksharing and parallel review programs: International work-sharing Australia – Canada – Singapore –Switzerland – UK (Access) Consortium, and the Project Orbis with the U.S. FDA. To facilitate information sharing, recognition and reliance activities, confidentiality agreements and Memorandums of Understanding between the NRAs are utilised.

### Standard Pathway

The standard pathway is available for products which do not meet the criteria for PR or PA. The pathway has not changed as a result of the MMDR review reform and detailed guidance is available on the TGA website [20]. The time for approval in the standard pathway has been steadily declining in the past decade, with a median time of 330 days for approval of new active substances in 2020 (from submission until approval, inclusive of NRA and company time) [9].

### Priority Review (PR)

The PR pathway is available for new prescription medicines or new indications for seriously debilitating or life-threatening conditions [17]. The level of clinical evidence required for priority medicines is identical to that of the standard review pathway, that is, a full dossier and substantial evidence of safety and efficacy [21]. The TGA target timeframe for approval is 150 working days, excluding the time required for the sponsor to answer TGA questions. A pre-submission meeting with the TGA is highly recommended. A PR determination is required before the full dossier is submitted to the TGA and approved determinations are published on the TGA website [22]. The TGA offers guidance on the PR determination and registration processes on their website [20]. Once determination has been granted by the TGA, the application for registration for a specific medicine (active ingredient) and priority indication may proceed. Any additional indications are subject to a separate application. The PR pathway results in full registration on the ARTG [23]. PR is not linked to orphan drug designation, with orphan designation being separate and non-mutually exclusive. However, where priority determination is for an identical medicine and indication (or a subset of indication) as the orphan designation, applications for determination/designation can be submitted simultaneously with joint justification for certain eligibility criteria [23, 24]. PR and PA are mutually exclusive.

### Provisional Approval (PA)

The PA pathway is intended for promising new prescription medicines or new indications for such medicines, for seriously debilitating or life-threatening conditions, where preliminary clinical evidence shows a potential to address an unmet clinical need or significant improvement over existing therapies [24, 25]. A pre-submission meeting with the TGA is highly recommended. A PA determination is required before the full dossier is submitted to the TGA. Approval is granted based on preliminary clinical data and results in a provisional (not standard and full) registration, time limited to 2 years, with an option for two further two-year extensions, to a total of 6 years. Conditions of registration may be re-assessed and changed at each extension point [16]. Before this validity period lapses, the applicant must provide comprehensive clinical evidence required for full registration. Despite its indication for serious diseases, the target assessment time is the legislated timeframe of 255 working days. However, the TGA aims to prioritise provisional medicines within the timeframe [17, 26]. The registration is conditional on provision of a clinical study plan, as part of the Risk Management Plan (RMP), outlining planned confirmatory data collection within a maximum of 6 years post registration. Unlike PR, PA may include submission of overseas evaluation reports during the assessment phase and after the provisional registration. The only scenario in which the provisional registration can be extended beyond 6 years is where an application is made for a full registration on the ARTG whilst the goods are still provisionally approved. Whilst such an application is in review, provisional registration remains valid [16]. An application for orphan drug designation may be submitted at the same time as the PA determination request, if the product fulfils the orphan drug criteria [23, 24].

To remain eligible for the provisional registration, the benefit-risk analysis of the medicine must be assessed on a regular basis and remain positive. The sponsor must complete confirmatory studies and provide the safety and efficacy data to the TGA in accordance with the RMP and the clinical trial plan timelines, both imposed as a condition of provisional registration.

Apart from the standard pharmacovigilance and RMP update requirements, the TGA uses an enhanced post-market monitoring and compliance framework, and prioritises provisionally approved medicines for post-market surveillance activities. Additional requirements can be imposed on a case-by-case basis, including collection of safety data using an Australian patient registry, enhanced communications to health care professionals and patients, more frequent or more prolonged submission of Periodic Benefit-Risk Evaluation Reports (PBRER) and possible selection of product for TGA laboratory testing. All PA medicines are included in the Black Triangle Scheme, encouraging increased vigilance and adverse event reporting. Under this scheme, a black triangle symbol appears on the Consumer Medicines Information (CMI), Product Information (PI) and certain TGA materials, such as the Australian Public Assessment Report (AusPAR).

Concerns have been raised in other jurisdictions regarding the heterogenous quality of evidence leading to approvals via expedited or conditional approval pathways, sponsor non-compliance with post-approval requirements and low quality of post-approval trials where these were conducted per requirements [27, 28]. In Australia, the TGA has indicated a commitment to closely monitor PA products and ensure fulfilment of the sponsor commitments via the RMP compliance monitoring program. The TGA will notify the sponsor where high priority activities are not delivered within timelines. Non-compliance may result in regulatory action such as penalties, suspension or cancellation of the provisional registration [29].

A comparison between the PR, PA and standard pathways is provided in Table 2.

**Table 2:** Comparison of Certain Elements of Standard and Facilitated Pathways [21, 22, 25, 28–31, 39–41]

	Priority Review	Provisional Approval	Standard
Determination eligibility criteria	-New medicine or new indication medicine AND; -Diagnosis, prevention or treatment of seriously debilitating or life-threatening condition AND; -No alternative goods registered for the condition (provisionally registered goods excluded) OR the priority product is a substantial improvement in safety or efficacy AND; -Major therapeutic advance	-New medicine or new indication medicine AND; -Diagnosis, prevention or treatment of seriously debilitating or life-threatening condition AND; -No alternative goods registered for the condition (provisionally registered goods excluded) OR the provisional product is a substantial improvement in safety or efficacy AND; -Preliminary data demonstrates major therapeutic advance AND; -Clinical study plan addressing submission of comprehensive safety and efficacy data within 6 years post registration	Not applicable
Recommended timing of submission for determination	3 months prior to submission for registration	3 months prior to submission for registration	Not applicable
Determination review target timeframe	20 working days (excluding time for sponsor reply to TGA queries)	20 working days (excluding time for sponsor reply to TGA queries)	Not applicable
Determination validity period	6 months; no extension	6 months initial period, with possible single extension of further 6 months	Not applicable
Pre-submission Planning Form (PPF)	Processing starts upon receipt of PPF	Processing same as in Standard process	Processing starts on the 1^st^ of a month
Submission for registration	-Lodge as soon as TGA identifier is available following PPF submission -eCTD format only	-Lodge as soon as TGA identifier is available following PPF submission -eCTD format only -Submission must be lodged within 6 months post notification of positive determination outcome -Submissions are batched as in the Standard process	-Following PPF submission, TGA evaluates the form and if PPF is acceptable, issues a Planning letter -Lodge submission after receiving the Planning letter (Milestone 1), by the date specified in the letter -Submissions are batched for processing
Preliminary application assessment	Yes (Milestone 2, acceptance of application for registration into the pathway) -40 working days	Yes (aka Milestone 2, acceptance of application for registration into the pathway) -40 working days	Yes (Milestone 2, acceptance of application for registration into the pathway) -40 working days
Review duration legislative timeframe	255 working days; target timeframe 150 working days (excluding time for sponsor reply to TGA queries)	255 working days (excluding the time required for sponsor reply to TGA queries), however TGA will prioritise provisional medicines within the timeframe	255 working days (excluding the time required for sponsor reply to TGA queries)
Review phases and milestones	-8 phases, each resulting in a milestone. Phases are “dynamic” -8 milestones, only milestone 2 (submission acceptance & clock start) and 7 (decision & clock stop) are formal	-8 phases -Milestone 1 (Planning letter) is not formal	-8 phases, each resulting in a milestone -8 milestones
Rolling questions	Yes	No	No
Rolling data submission	No	Yes; scope and timing prospectively agreed and confirmed between sponsor and TGA at pre-submission meeting, determination application and submission points. Applicable to data affecting decision	No
Response to consolidated TGA request for information under Section 31 of the Therapeutic Goods Act 1989	Sponsor commits to respond within 30 days	Sponsor can choose to respond within 30 days or 60 days	Sponsor can choose to respond within 30 days or 60 days
Contact with TGA	Pre-submission meeting (optional), up to 3 months prior to determination application and 3–6 months prior to submission for registration	Pre-submission meeting strongly recommended. 3 months prior to determination application and 6 months prior to submission for registration	Pre-submission meeting prior to PPF or application lodgement (optional). Recommended for complex applications

### Comparable Overseas Regulator Pathways

Following the MMDR review, the TGA developed a Comparable Overseas Regulator (COR) report-based submission process with a set of criteria for NRAs to be considered a comparable regulator and for the submission package to be suitable for this pathway. The pathway requires a full dossier as submitted to the COR NRA and an Australian specific Module 1, all in eCTD format [13, 30]. As per these criteria, a comparable NRA would have a regulatory framework and scope of work similar to the TGA, with assessment reports and cooperation communications in English. It would use international standards and guidelines consistent with those adhered to by the TGA and have a Memorandum of Understanding (MOU) or other arrangement in place with the TGA to facilitate cooperation and information sharing. The TGA currently considers the following seven NRAs comparable, based on existing collaboration arrangements: FDA, EMA, Medicines and Healthcare products Regulatory Agency (MHRA, UK), PMDA, Health Canada, Health Sciences Authority (HSA, Singapore), and Swissmedic [30].

For a COR submission package to be accepted, its reports must be unredacted, complete and include advisory body correspondence and advice for a de novo evaluation, in CTD format. The submission package should be the same and the indication equivalent in terms of dosage, population profile and patient outcomes. The TGA reserves the right to use COR report information in the AusPAR, subject to restrictions applicable to any Australian submission [30]. Pre-submission meetings are encouraged to discuss any dissimilarities between the COR and TGA submissions and processes [30].

**Figure 2:**
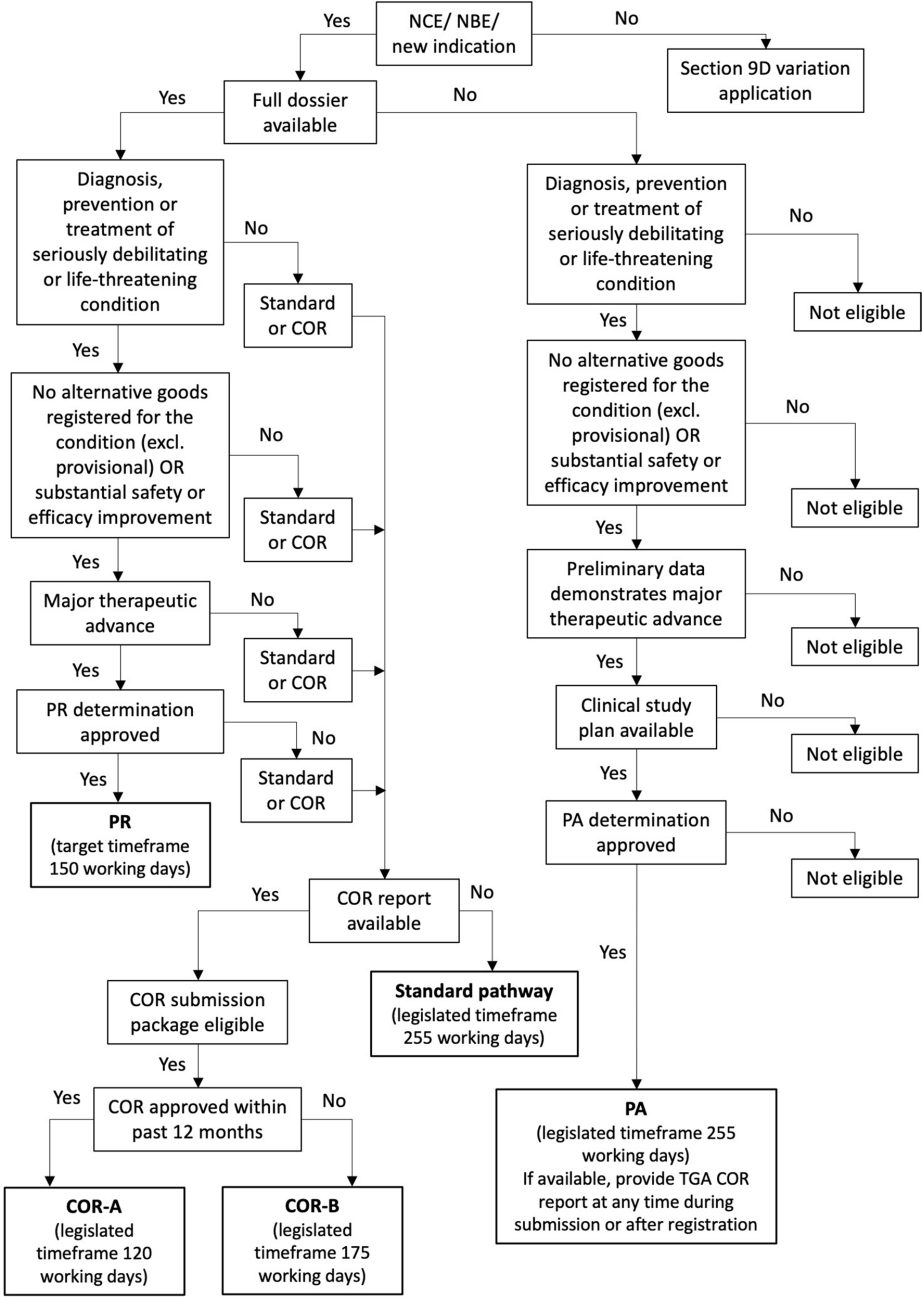
TGA pathway selection map

The COR process is suitable for both new prescription medicines and variations, where a complete, de novo assessment by a COR has resulted in full marketing approval, with no deferral, rejection or withdrawal anywhere in the world at any time [30]. The COR-A legislated timeframe is 120 working days [17]. For this pathway, the COR approval must be within the last 12 months, for the same medicine and manufacturing conditions. The TGA assessment is then limited to Australia-specific documentation such as the Product Information (PI), labelling and RMP. The COR-B legislated timeframe is 175 days [17]. This pathway is suitable for cases where assessment of data is needed in addition to the PI, label and RMP. There is no limit on the COR approval timing, and it is understood that for older approvals changes may have occurred in both the regulatory processes and medicine related aspects. Sponsors should consider the merits of each of these pathways in their regulatory strategy decisions and Fig. 2 provides a roadmap to support registration pathway selection.

### International Work Sharing - Access Consortium

This initiative commenced in 2007 between the TGA, Health Canada, Swissmedic, and HSA of Singapore with the aim to increase regulatory reliance and information sharing, exchange knowledge and technical expertise, minimise duplication of regulatory effort, improve efficiency and resource allocation and ultimately ensure timely access for patients [19]. The MHRA, the UK NRA, joined in October 2020. In Australia eight products were approved via this initiative between July 2018 and May 2020: five oncology products (apalutamide, abemaciclib, niraparib, darolutamide and isatuximab), two for a cardiology indication (tafamidis and tafamidis meglumine) and baloxavir marboxil indicated for the treatment of influenza. Whilst the average number of TGA working days for standard pathway NCE assessment in 2018–2020 was 197 (calculated from commencement of evaluation until approval, TGA working days, i.e. NRA time only), the average time to approval via the Access Consortium was 161 days, highlighting the benefits for patient access to new medicines using this pathway [31].

This unique international work-sharing arrangement applies to submissions filed for joint review in more than one Access jurisdiction. Duplication of sponsors’ efforts is minimised due to common dossier submission and combined requests for information. Marketing authorisation in the jurisdictions occurs concurrently, with each NRA making an independent sovereign decision. An Access submission may be made via the standard or PR pathway (same pathway in all jurisdictions), as agreed between the participating NRAs [19].

### Project Orbis

TGA has become a partner with the US FDA Oncology Center of Excellence (OCE) Project Orbis initiative since May 2019. Project Orbis provides a pathway for oncology submissions for parallel collaborative review by evaluating NRAs. The initiative is intended for high impact oncology products which meet the criteria for FDA Priority Review. Participating NRAs are TGA, FDA, Health Canada, HSA, Swissmedic, ANVISA (Brazil), MHRA and Israel. The first Project Orbis evaluation was jointly completed by the FDA, TGA and Health Canada, resulting in simultaneous approval on 17 September 2019 for a combination treatment (lenvatinib and pembrolizumab) for advanced endometrial cancer. In the first year of operation 60 applications were submitted for new molecular entities or new indications, resulting in 38 approvals [32]. In Project Orbis there are now three types of applications which differ in the timelines of submission and associated regulatory action to FDA and participating partners (regular, modified or written report). For Type A submissions (Regular Orbis), the dossiers are lodged concurrently to participating NRAs. During the concurrent evaluation each NRA adheres to their own regulatory process and uses a pathway equivalent to the FDA priority review pathway. Information request responses are provided to each Project Orbis partner. Decision making is independent. The project offers flexible approach and allows Type B (Modified Orbis) and Type C (Written Report Only) collaboration avenues. Type B is suitable for applications submitted with over a 30-day delay or a regulatory action more than 3 months of the FDA action. Type B allows only concurrent review with FDA, however no concurrent action. Type C application is suitable where FDA has already taken a regulatory action and the report can be shared with the Orbis partner(s). This avenue does not allow concurrent review or action [18, 33, 34].

In Australia, lenvatinib/pembrolizumab (PA, 54 TGA working days), acalabrutinib (PA and utilising COR-B, 35 working days), nivolumab/ipilimumab (68 working days), ripretinib (PR, 123 working days) and tucatinib (PR, 113 working days) have been approved under this process by the end of 2020. The evaluations via Project Orbis have been completed within very impressive timeframes, median time to approval by FDA was 4.2 months and by Orbis partners 4.4 months. OCE’s Real - Time Oncology Review (RTOR) pilot program, used in Project Orbis, may be a contributing factor. RTOR involves provision of data to FDA prior to submission for registration. This allows the FDA to evaluate the data early, provide feedback to the sponsor and address potential issues, and thus may facilitate a quicker, streamlined and more efficient assessment process once the submission occurs. The FDA can also share their scientific and technical assessments earlier for TGA and other NRAs to target their review questions.

## Industry Adoption of FRPs

**Figure 3:**
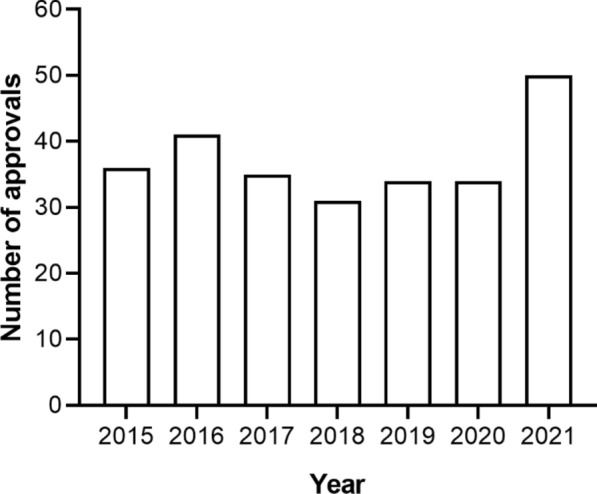
Number of NCEs approved by the TGA between 2015 and 2021 [36]

**Figure 4:**
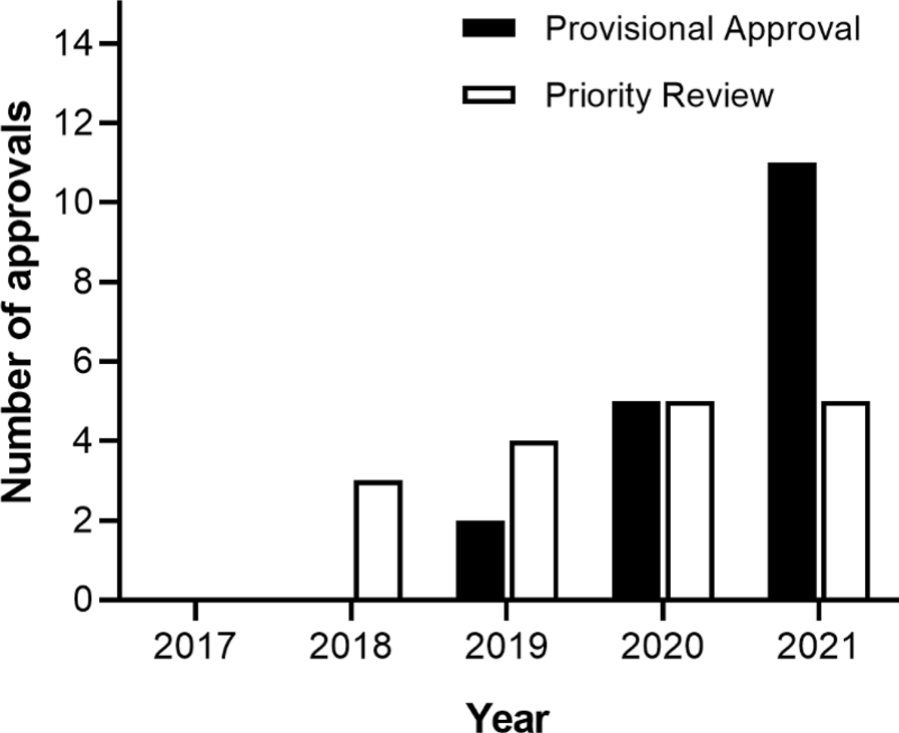
Number of TGA Priority Review and Provisional Approvals 2017–2021 [36]

**Figure 5:**
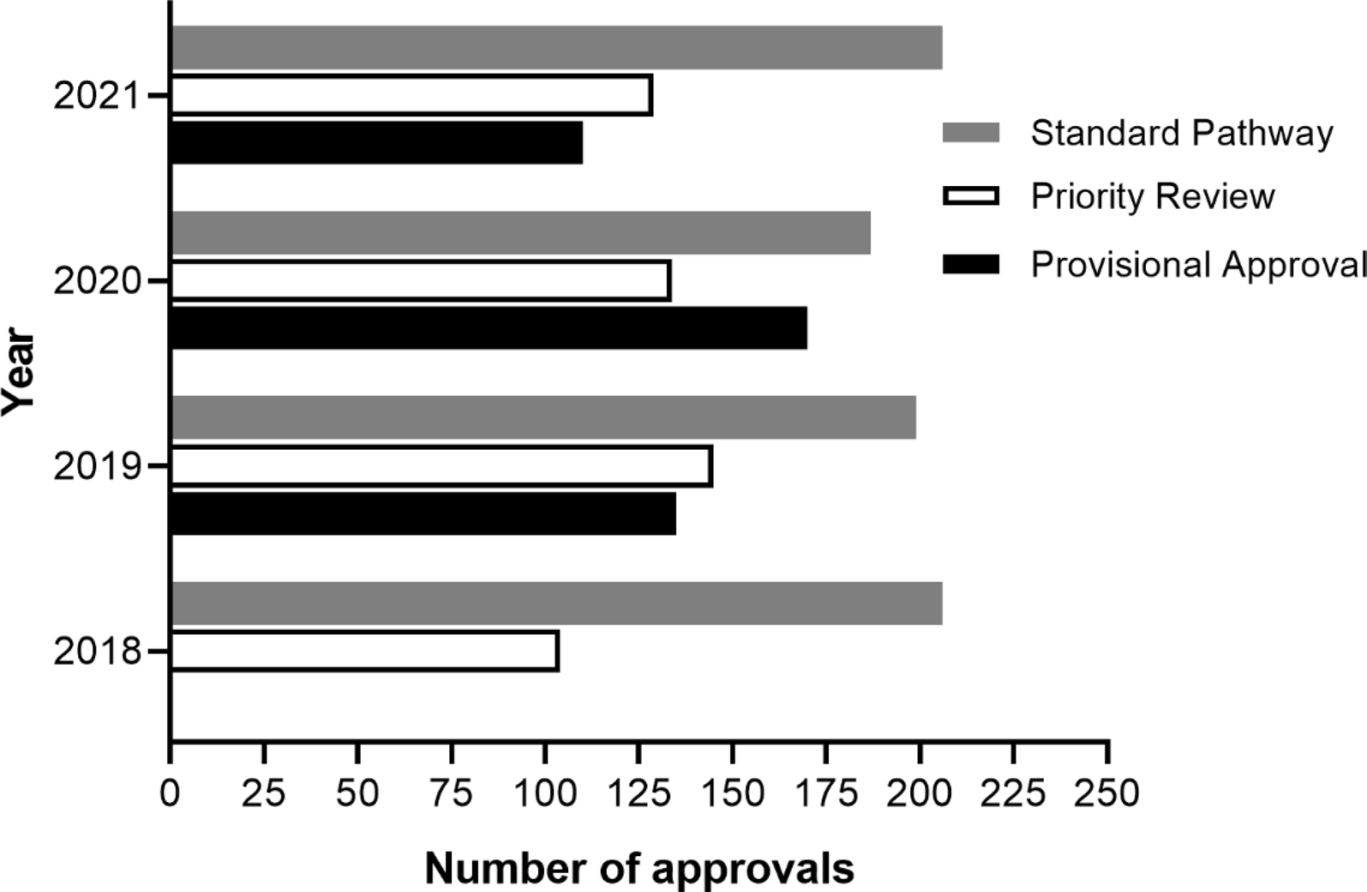
Mean time to approval in Priority Review, Provisional Approval and standard pathways in 2018–2021 [36]

**Figure 6:**
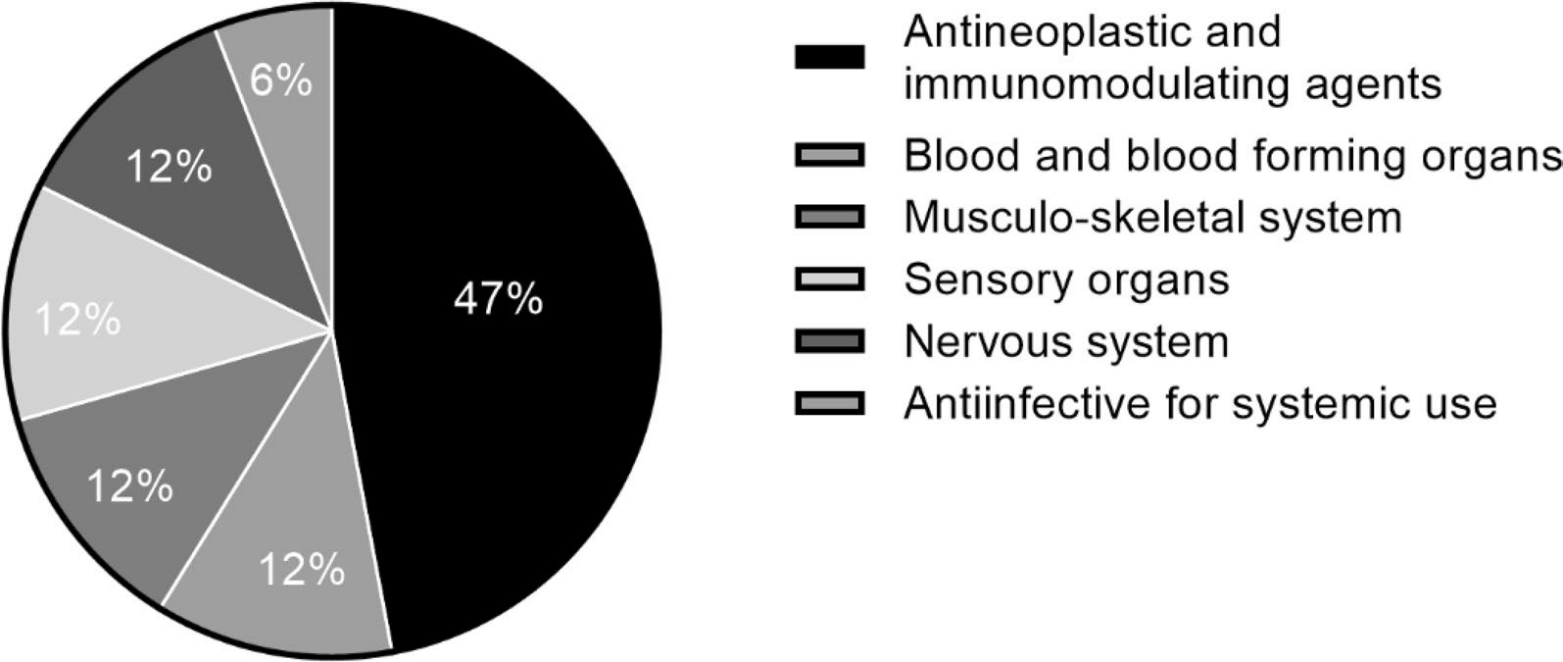
ATC classification of NCEs approved via Priority Review in 2018–2021 [36]

**Figure 7:**
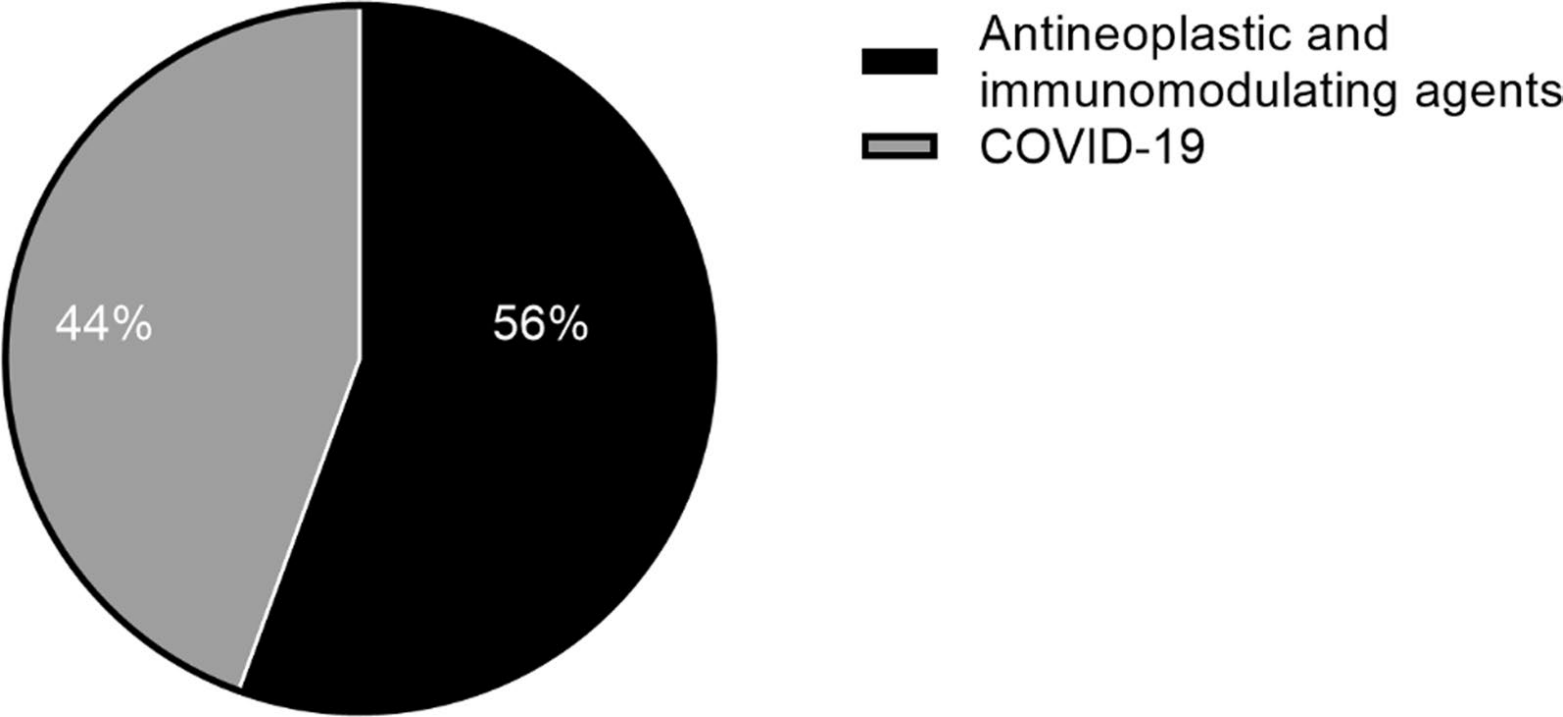
ATC classification of NCEs approved via Provisional Approval in 2019–2021 [36]

The TGA approved approximately 35 NCEs annually between 2015 and 2020, with an increase to 50 NCEs in 2021 (Fig. 3) [31]. With PR availability since July 2017 and PA availability since March 2018, the number of NCE medicines registered via these pathways is gradually increasing from three to five and from two to eleven, respectively (Fig. 4). The mean time to approval in the FRPs, calculated in TGA working days, has been shorter than the approval time for medicines utilising the standard pathway (Fig. 5) [31]. In both FRPs, the most common indication, classified using the World Health Organisation Collaborating Centre for Drug Statistics Methodology Anatomical Therapeutic Chemical classification, was oncology, i.e. antineoplastic and immunomodulating agents, as shown in Fig. 6 for PR and Fig. 7 for PA [31].

**Figure 8:**
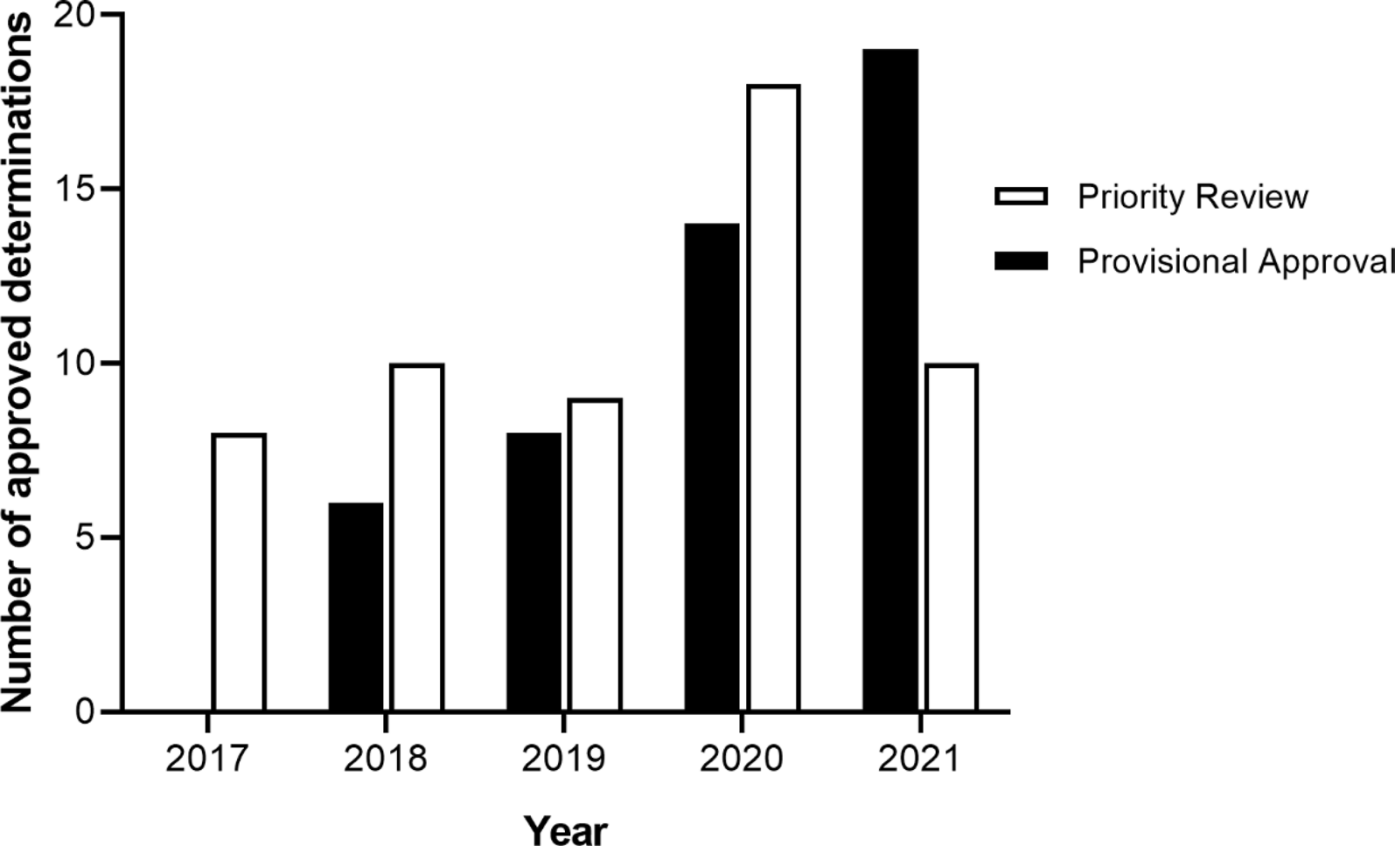
Number of approved Priority Review and Provisional Approval determinations 2017–2021 [42]

**Figure 9:**
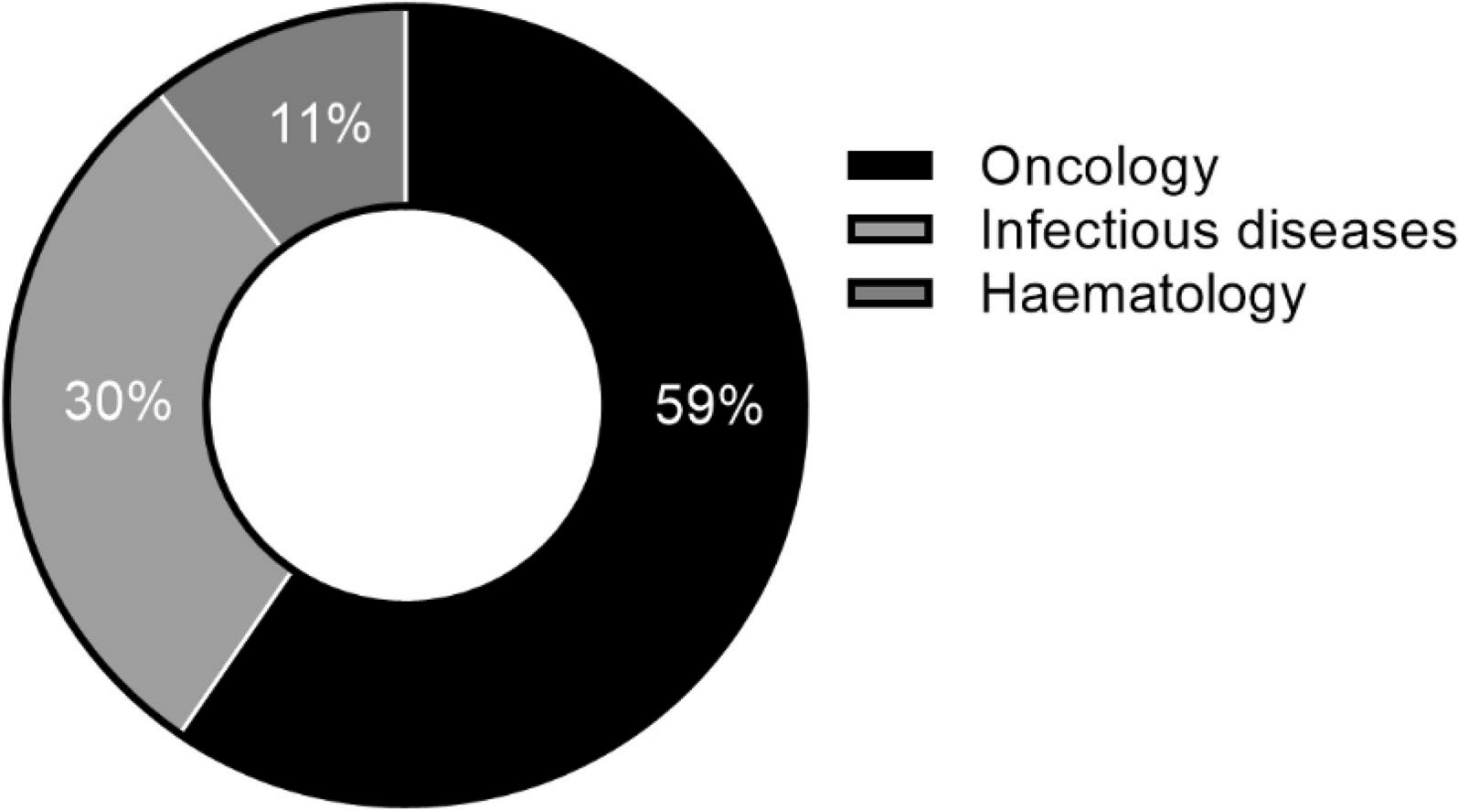
Provisional approval determinations 2018–2021: therapeutic area [42]

**Figure 10:**
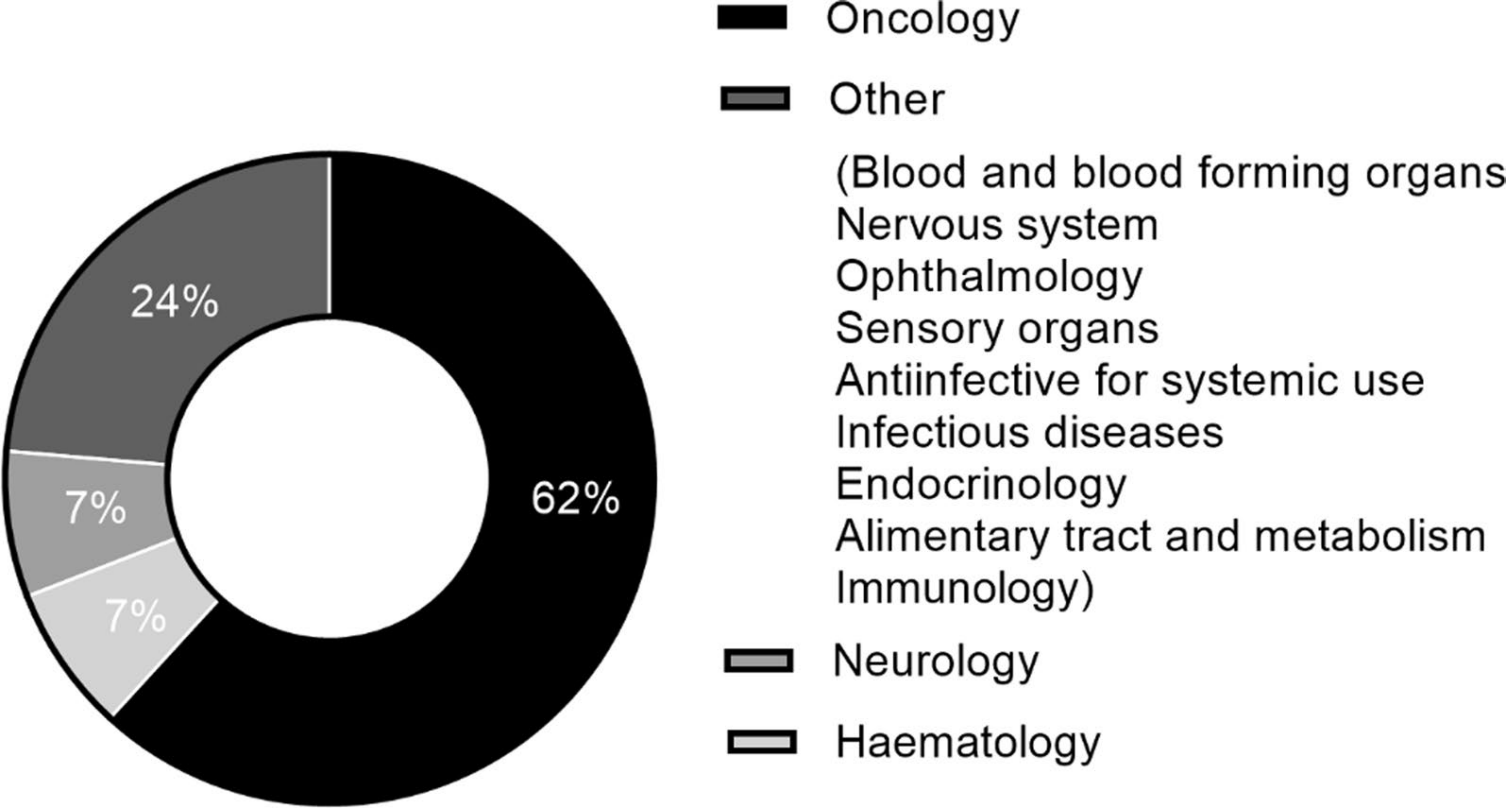
Priority Review determinations 2017–2021: therapeutic area [42]

From the time of a FRP becoming available in 2017 until the end of 2021, the TGA had granted 55 PR determinations and 47 PA determinations. There was a marked increase in the number of determinations approved under both pathways in 2020, with the number of PA determinations continuing to increase in 2021 (Fig. 8). The most frequent therapeutic area for determinations is oncology in both PA (59.6%) and PR (61.8%), followed by infectious diseases in PA (Fig. 9), haematology and neurology in PR (Fig. 10). The increase in applications demonstrates that the industry is interested in and utilising these new registration options. For instance, in 2021 the PA was utilised extensively for COVID-19 products, resulting in a significant increase of the number of products going through the pathway—from five in 2020 to eleven, of which seven were for COVID-19 related indications. The shorter mean time to approval for PA in 2021 (110 TGA working days) was also affected by the seven COVID-19 related products, which had an average approval time of 60.7 TGA working days.

## Discussion

Australia has undergone a recent comprehensive review of its regulatory system for therapeutic products. The MMDR review strengthens parameters considered to be key hallmarks of a strong regulatory system, particularly with respect to regulatory convergence, reliance, recognition and technical harmonisation [35]. The review and resulting guidelines development have been conducted in a transparent and open manner with repeated public consultations and inputs from a wide range of stakeholders, including industry, healthcare professionals, professional bodies, consumer organisations and government. This helped ensure the guidelines addressed relevant and significant issues identified by the stakeholders, and therefore were fit for purpose [2].

The resulting FRP review processes have clearly defined milestones and timelines for both the regulator and the applicant, with a preliminary assessment stage which allows filtering of applications which do not meet the requirements and prevents time wastage. Communication from the TGA to the applicant is written into the process, keeping the applicant informed on the progress and planned future steps. This is a way to ensure that TGA review remains highly predictable with low variability of review times. The TGA has developed a clear application prioritisation mechanism, with transparent criteria for products with the highest expected benefit. The review timelines, determination decisions and final registration decisions are transparent and publicly available. The TGA publishes positive outcomes on both the determination and registration decisions on their website. An AusPAR is also publicly available, outlining their decision-making process and rationale [36].

As a result of the reform, the TGA now has the required arsenal of review mechanisms for prescription medicines, which enables flexibility and appropriateness of review methods and timelines. The two newly introduced FRPs allow either reduced evaluation times or product availability to consumers at an earlier stage in the clinical development process. New registration pathways have been a welcome addition and are being actively utilised by the industry. During the COVID-19 pandemic, when a flexible approach and speedy access were needed, these pathways have provided unprecedented benefits to the sponsors who were able to bring their COVID-19 indicated medicines into Australia and have them approved and reimbursed in a timely manner and to the Australian consumer. The capability, enabled by the pathways, for TGA to expedite the review and utilise collaboration, reliance and recognition mechanisms, was instrumental in this unparalleled situation.

In recent years the trend towards regulatory convergence and harmonisation between NRAs has been growing [1–4]. Harmonisation may be defined as the process of integrating national and international standards to facilitate efficiency in global drug development and regulation, whilst convergence may be defined as the process whereby the regulatory requirements across countries become more aligned over time as a result of adoption of these international standards [5].

This trend is instrumental in optimising the registration process for a new medicine in each individual country in terms of time, efficiency and quality, with minimal duplication of resources, time or cost. An essential component and principle enabling such activities is trust between NRAs, which is based on consistency of scientific review and comparability of frameworks and is a primary factor allowing for greater scientific collaboration. The evaluation work undertaken by multiple NRAs is often based on the same data set and the sponsor may have to repeatedly address similar questions posed by multiple NRAs. This results in duplication of regulatory efforts, prolongation of evaluation times and suboptimal allocation of limited resources [4, 6]. Additionally, medicines entering the market are becoming more complex, such as biologicals, cell-based and gene therapies, and this is further precipitating the skill shortage amongst NRAs to conduct comprehensive reviews of these products. Greater harmonisation, convergence and work-sharing between NRAs is one way to address these constraints. As part of the reform, pathways using overseas evaluation reports have been revised. The reform has strengthened the already existing collaboration mechanisms such as the Access consortium and expanded them as in the case with the COR-A and COR-B pathways. Future research is required to further assess the long-term impact of the MMDR review changes, as well as determine the stakeholder perspective on the regulatory landscape to ensure Australia’s regulatory system remains fit-for-purpose into the future.
